# Land management explains major trends in forest structure and composition over the last millennium in California’s Klamath Mountains

**DOI:** 10.1073/pnas.2116264119

**Published:** 2022-03-14

**Authors:** Clarke A. Knight, Lysanna Anderson, M. Jane Bunting, Marie Champagne, Rosie M. Clayburn, Jeffrey N. Crawford, Anna Klimaszewski-Patterson, Eric E. Knapp, Frank K. Lake, Scott A. Mensing, David Wahl, James Wanket, Alex Watts-Tobin, Matthew D. Potts, John J. Battles

**Affiliations:** ^a^US Geological Survey, Menlo Park, CA 94025;; ^b^Department of Environmental Science, Policy, and Management, University of California, Berkeley, CA 94720;; ^c^Department of Geography, Geology and Environment, University of Hull, Hull HU6 7RX, United Kingdom;; ^d^The Yurok Tribe’s Cultural Resources Manager, Klamath, CA 95548;; ^e^USDA Forest Service, Deschutes National Forest, Bend, OR 97701;; ^f^Department of Geography, California State University, Sacramento, CA 95819;; ^g^Pacific Southwest Research Station, USDA Forest Service, Redding, CA 96002;; ^h^Pacific Southwest Research Station, USDA Forest Service, Arcata, CA 95521;; ^i^Department of Geography, University of Nevada, Reno, NV 89557;; ^j^Department of Geography, University of California, Berkeley, CA 94720;; ^k^The Karuk Tribe’s Department of Natural Resources, Orleans, CA 95556

**Keywords:** Indigenous management, forest biomass, restoration, carbon policy, land use

## Abstract

For millennia, forest ecosystems in California have been shaped by fire from both natural processes and Indigenous land management, but the notion of climatic variation as a primary controller of the pre-colonial landscape remains pervasive. Understanding the relative influence of climate and Indigenous burning on the fire regime is key because contemporary forest policy and management are informed by historical baselines. This need is particularly acute in California, where 20th-century fire suppression, coupled with a warming climate, has caused forest densification and increasingly large wildfires that threaten forest ecosystem integrity and management of the forests as part of climate mitigation efforts. We examine climatic versus anthropogenic influence on forest conditions over 3 millennia in the western Klamath Mountains—the ancestral territories of the Karuk and Yurok Tribes—by combining paleoenvironmental data with Western and Indigenous knowledge. A fire regime consisting of tribal burning practices and lightning were associated with long-term stability of forest biomass. Before Euro-American colonization, the long-term median forest biomass was between 104 and 128 Mg/ha, compared to values over 250 Mg/ha today. Indigenous depopulation after AD 1800, coupled with 20th-century fire suppression, likely allowed biomass to increase, culminating in the current landscape: a closed Douglas fir–dominant forest unlike any seen in the preceding 3,000 y. These findings are consistent with precontact forest conditions being influenced by Indigenous land management and suggest large-scale interventions could be needed to return to historic forest biomass levels.

## Significance

We provide the first assessment of aboveground live tree biomass in a mixed conifer forest over the late Holocene. The biomass record, coupled with local Native oral history and fire scar records, shows that Native burning practices, along with a natural lightning-based fire regime, promoted long-term stability of the forest structure and composition for at least 1 millennium in a California forest. This record demonstrates that climate alone cannot account for observed forest conditions. Instead, forests were also shaped by a regime of frequent fire, including intentional ignitions by Native people. This work suggests a large-scale intervention could be required to achieve the historical conditions that supported forest resiliency and reflected Indigenous influence.

Fires ignited by lightning and Indigenous people have influenced the structure and composition of forest ecosystems in the American West for millennia (1, 2). In California, Indigenous knowledge from tribal sources, historical ethnographic accounts by Euro-Americans, and ecological reconstructions all document landscape features consistent with a regime of frequent fire consisting of both lightning and Indigenous origin (3, 4). However, the extent of the impact of Indigenous burning on Californian ecosystems continues to be contested. Some posit that climate and climatically induced factors (e.g., a fire regime) were the major determinants of forest dynamics (5, 6); others argue that Indigenous burning was a major driver of ecosystem structure and composition (7, 8). Inaccurate assessments of Indigenous fire use on past landscapes may generate misleading inferences about the best way to conserve fire-prone ecosystems (9). Thus, characterizing the effect of Indigenous stewardship on ecosystems—an Indigenous baseline—is a pressing scientific challenge with important implications for contemporary land management (10–12).

Over the past century, California’s fire-prone conifer forests have been altered by the curtailment of Indigenous stewardship, the exclusion of fire, and the harvest of merchantable trees (13, 14). While the relative importance of these factors varies geographically, their impact has been widespread. The forests’ management history, coupled with a warming climate, places them at risk from large, high-severity fires that threaten both the forests and the communities that depend on them (15, 16). Wildfire hazards also jeopardize the state of California’s plan to manage forest ecosystems for carbon storage as part of its climate mitigation efforts (17). Successful management and conservation efforts partly depend on meaningful comparisons between modern conditions and long-term histories (18), as well as an understanding of how humans have shaped historical baselines (10).

As such, California forests provide a highly pertinent setting to test for human-modified baselines with the goal of transferring information to land managers, particularly given the state’s explicit recognition of the importance of historical perspectives (19). However, current wildfire and resiliency discussions still lack an understanding of how recurring fire impacted forest conditions over long time horizons. An accurate reference of ecosystem dynamics requires incorporation of data predating Euro-American colonization. In this study, we seek to accurately document the Indigenous baseline for forests in the Klamath region of California. Specifically, we ask: Can climate alone explain major trends in reconstructed forest biomass over the past 3,000 y?

In answering this question, we provide a blueprint for integrating multiple forms of paleoevidence, each with its own strengths and weaknesses, to provide a transdisciplinary record of land management using two watersheds in the western Klamath Mountains, CA (20). Previous research has separately presented paleoecological and ethnographic information (12, 21–23) to argue for the preeminence of human-caused vegetation change over climatically driven vegetation changes in California. For example, paleoecological evidence has suggested open-forest conditions and shade-intolerant vegetation when the prevailing climate favored the development of a closed-canopy forest with shade-tolerant vegetation (23). That is, the signal of a human-modified landscape was assumed when shade-intolerant taxa (e.g., *Quercus*) persisted during cool, wet periods even though shade-tolerant taxa (e.g., *Pseudotsuga*) should climatically dominate (e.g., the Little Ice Age [LIA] (23)).

**Fig. 1.**
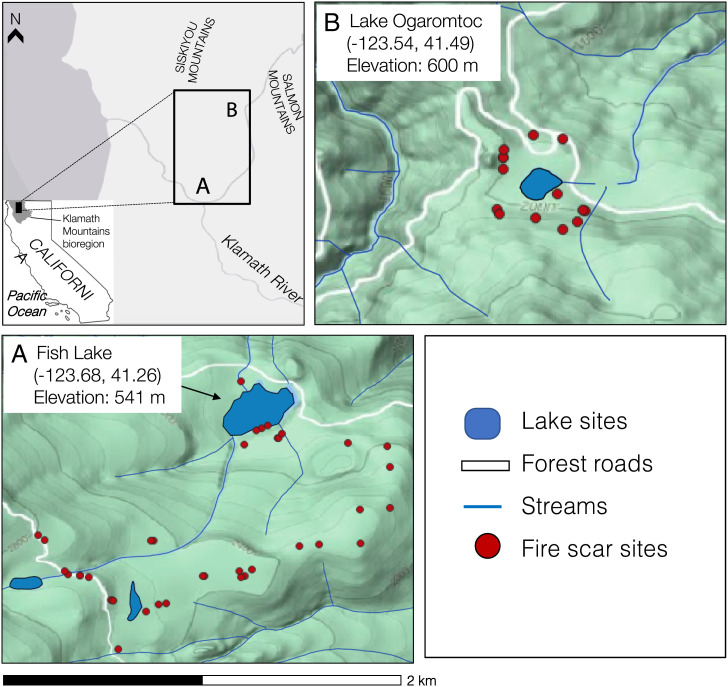
Map showing study sites in northwestern California and shaded-relief map of the surrounding watershed. Lakes and streams are shown in blue, while white lines indicate forest roads. Fire scar samples (red circles) were taken near Fish Lake and Lake Ogaromtoc in 2008 to 2010. Cores were taken from (*A*) Fish Lake in 2008 and (*B*) Lake Ogaromtoc in 2009.

We improve the evidentiary base using a mixed-methods approach that leverages the value of each individual proxy by relating it to the other information. We compiled data from multiple sources about Fish Lake and Lake Ogaromtoc (Fig. 1) (24), two lakes surrounded today by montane mixed hardwood-conifer forests with a diverse canopy of tree species (*SI Appendix*, Table S1) (25). Fish Lake is an area of joint use between the Karuk and Yurok Tribes, and Lake Ogaromtoc is a cultural-use site in the Karuk’s ancestral territory. The 3,000-y paleoecological record derives from sediment cores from these low-elevation lakes. These lakes provide an ideal setting to test the paradigm that climate controls vegetation change above and beyond human modification because of the diversity of available data, with implications for modern landscape management. Data are presented in decreasing order of detail and sensitivity of the indicator. We drew from existing Karuk and Yurok Tribe ethnographies (12) and Karuk/Yurok-based traditional ecological knowledge (*section 2.1*); paleoecological records (biomass, charcoal, and fire scars) (*section 2.2*); correlations among climate, vegetation, and fire proxies during climatically anomalous periods (*section 2.3*); and multiple independent cross-references (26) to validate our biomass record with historical data from AD 1880 onward (*section 2.4*).

## Results

### 2.1 Indigenous Burning Practices Resulted in Significant Landscape Modification.

Indigenous people have inhabited the Klamath-Siskiyou bioregion for at least 9,000 y (27). Oral histories from the 20th century about the previous two centuries, and tribal lore about stewardship and burning dating hundreds to thousands of years before present, suggest Indigenous people of the Klamath Mountains—the Karuk and Yurok—have intentionally ignited, and still ignite, fires for numerous reasons, including to produce food and fiber, to support ceremonial practices, to clear travel corridors, to promote hunting, and to reduce pest populations (2, 28, 29). The Karuk describe fire as “crucial to who people are, and what they do…enable[ing] them to live” (30).

The forest structure and composition encountered by Euro-American colonists in the mid-1800s was thus shaped by cultural burning practices developed over the last millennia (27). Specifically, in the watersheds encompassing Fish Lake and Lake Ogaromtoc, the routine deployment of small situational burns (i.e., patch burns of <10 ha) along with larger broadcast burns (i.e., >10 ha) during the late winter/early spring and late summer/early fall (31) altered the landscape. Patch burning is considered a targeted application of fire to fuels within a bounded area for specific purposes. In contrast, broadcast burning sets fire to the landscape for multiple purposes and with general boundaries constrained by topography (i.e., slope and aspect) and fuels (i.e., loads and connectivity). For the Yurok, the Fish Lake area was historically used for gathering hazelnuts, acorns, berries, and basket materials; hunting game; camping; and other subsistence and ceremonial practices (30; see *Materials and Methods*). The Karuk used Fish Lake and Lake Ogaromtoc for purposes similar to the Yurok, including as traditional gathering places for acorns from *Quercus kelloggii* (black oak) and *Notholithocarpus densiflorus* (tanoak), as well as mushrooms (31; see *Materials and Methods*).

Extensive trail systems from villages along the local rivers up to and beyond the lakes, such as the passageway along Rock Creek to Lake Ogaromtoc and along Bluff Creek to Fish Lake, facilitated tribal access. Patch burning was used to maintain these trails, reduce pests, and promote berry and root growth. Broadcast burning in the area facilitated habitat conversion, nut/acorn harvesting, and hunting (12). Burns could alter vegetation succession at timescales of decades to centuries (12, 32). In these watersheds, fire was applied in places where lightning fire was less common and in places to preempt lightning-ignited fires to achieve desired cultural conditions (33). These burning practices were concurrent with, and influenced by, two distinct climate events: the warm, dry Medieval Climate Anomaly (MCA, ∼1,200 to 850 calBP [calendar years B.P., where present is AD 1950]) and the cool, wet LIA (∼750 to 50 calBP) (34). For example, the greatest spatial extent of the Karuk’s burning occurred around 250 calBP to maintain fire-tolerant assemblages (including *Quercus* spp.) during the LIA that were promoted during the MCA’s more xeric conditions (35).

Karuk oral history also indicates the occurrence of structural change before and after Euro-American colonization. Lower fuel levels and open forest were critical to the cultivation of acorns, nuts, berries, mushrooms, and weaving materials (12, 36) that supported a population of at least 2,700 people circa AD 1860 (37). The modern forest, in contrast, is overstocked and underburned: “We never had this much fuel on the ground,” M. McCovey, a Karuk elder, said (12). Members of the Karuk and Yurok Tribes recognize that their traditional lands are overenriched in both live trees and woody debris; they characterize the current high-biomass forest conditions as a “degradation” of subsistence land (38; see *Materials and Methods*). Additional ethnographic and historical evidence is presented in *SI Appendix*.

### 2.2 Paleoecological Data Indicate Frequent Fire-Limited Biomass.

**Fig. 2.**
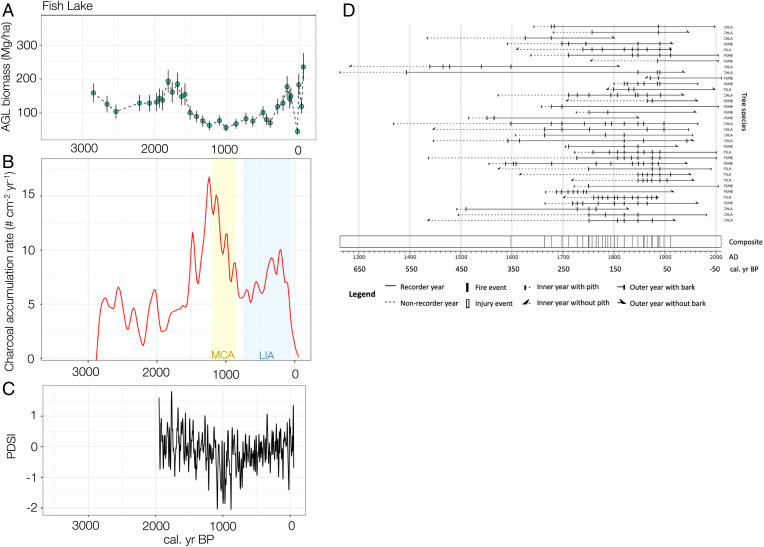
(*A*) Reconstructed AGL tree biomass (in megagrams per hectare, summation of major taxa, dots with SE bars) at Fish Lake between 2850 and −58 calBP. (*B*) Variation in charcoal influx (in particles per square centimeter per year), where the MCA is shaded yellow and the LIA is shaded blue. (*C*) Variation in PDSI reconstruction over time; data only extend to ∼2000 calBP. (*D*) Fire scar records for CHLA (*Chamaecyparis lawsoniana*), PILA (*Pinus lambertiana*), and PSME (*Pseudotsuga menziesii*), with the legend at the left; summary statistics are in Table 1. The composite record (*Bottom*) is based on fire events and filtered by the number of trees recording fires ≥ 2, with a minimum sample number ≥ 2.

**Fig. 3.**
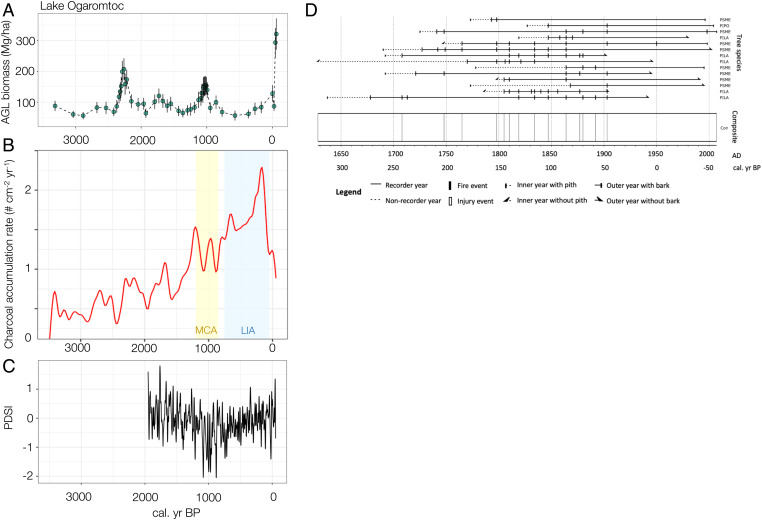
(*A*) Reconstructed AGL tree biomass (megagrams per hectare, summation of major taxa, dots with SE bars) at Lake Ogaromtoc between 3312 and −59 calBP. (*B*) Variation in charcoal influx (in particles per square centimeter per year), where the MCA is shaded yellow and the LIA is shaded blue. (*C*) Variation in PDSI reconstruction over time; data only extend to ∼2000 calBP. (*D*) Fire scar records for PILA, PIPO (*Pinus ponderosa*), and PSME, with the legend at the bottom; summary statistics are in Table 1. The composite record (*Bottom*) is based on fire events and filtered by the number of trees recording fires ≥ 2, with a minimum sample number ≥ 2.

Robust quantitative reconstruction of past plant abundance is possible using calibrated models of pollen influx (in grains per square centimeter per year) and aboveground live (AGL) tree biomass (in megagrams per hectare) (39). Using models parameterized with data from sites including Fish Lake and Lake Ogaromtoc, pollen influx values were transformed into AGL biomass using taxa-specific calibrated functions (39) and overlain with charcoal influx (in particles per square centimeter per year) and fire scar records from both lakes. Live biomass trends at both sites track proxies for fire occurrence, namely charcoal influx trends (Figs. 2 *A* and *B* and 3 *A* and *B*) and fire scar records (Figs. 2*C* and 3*C*). The fire scar record for Fish Lake and Lake Ogaromtoc indicates frequent fire (Table 1). For the period of maximum sample depth (1700 to 1900, ≥2 scars per site), the median return interval was 7 y for Fish Lake and 12 y for Lake Ogaromtoc. More samples were available at Fish Lake (*n* = 35) compared to Lake Ogaromtoc (*n* = 14). Most of the fire scars (Fish Lake = 91%, Lake Ogaromtoc = 81%) were recorded in the latewood or dormant position of the intra-annual tree ring, implying that the majority of burns impacting trees occurred in or after the late summer or fall.

**Table 1.** Summary of fire scar history at Fish Lake and Lake Ogaromtoc

				Median fire return interval (all years)		Median fire return interval (1700–1900)	
Site	No. of samples	Earliest scar	Last scar	All scars	≥2 scars for site	All scars	≥2 scars for site
Fish Lake	35	1393	1943	5	8	3	7
Lake Ogaromtoc	14	1678	1998	6	15	6	12

	Intraring scar position						
	No. of fire scars	Scars with position	Early earlywood (%)	Middle earlywood (%)	Late earlywood (%)	Latewood (%)	Dormant (%)
Fish Lake	172	107	0.9	2.8	5.6	52.3	38.3
Lake Ogaromtoc	77	53	3.8	7.5	7.5	49.1	32.1

Median fire return intervals are presented at two time periods and with different scar thresholds. Intraring scar positions are also summarized.

Despite variation over time and between sites, the charcoal and biomass records document more fire activity and lower live tree biomass in the past 3,000 y than in the contemporary forests. At Fish Lake, charcoal influx was relatively low, and biomass was above 150 Mg/ha before 1900 calBP. Between 1500 and 650 calBP, biomass dropped and remained under 100 Mg/ha, coincident with large increases in charcoal influx from 1500 to 900 calBP. Biomass started rising in 600 calBP; increased between 400 and 200 calBP, rising above 150 Mg/ha; and then abruptly dropped, consistent with increasing charcoal influx and multiple fire scars from 250 calBP onward. Several rapid increases and decreases in biomass occurred in the 20th century (see *section 2.4*). The highest predicted biomass values in Fish Lake’s record occur in the present and are over 250 Mg/ha, which is accompanied by sharply declining charcoal influx. During the baseline period of ∼2800 to 100 calBP, median tree biomass was 128 Mg/ha and the interquartile range (IQR) was 83 to 144 Mg/ha (*SI Appendix*, Fig. S1*A* and Table S2).

Unlike Fish Lake, biomass at Lake Ogaromtoc was estimated to have been under 150 Mg/ha prior to Euro-American contact, except during 200-y intervals around 2300 calBP and around 1100 calBP. Biomass fluctuated but generally remained around 100 Mg/ha from 2200 to 1200 calBP, during which time charcoal influx gradually increased. Charcoal influx increased throughout the MCA and LIA until the modern period, when it sharply declined. Fire scars from 250 to 50 calBP track increasing charcoal influx from 250 to 100 calBP and generally low biomass values. The abrupt cessation of fire events in the last century coincided with rising biomass exceeding 300 Mg/ha by 2008, despite an abrupt drop and recovery between the 1950s and 1970s that coincided with nearby logging activities (see *section 2.4*). Median tree biomass was 104 Mg/ha (IQR 87 to 113) between ∼3300 and 150 calBP (*SI Appendix*, Fig. S1*B* and Table S2).

### 2.3 Correlations Suggest Anthropogenic Burning during the LIA.

**Fig. 4.**
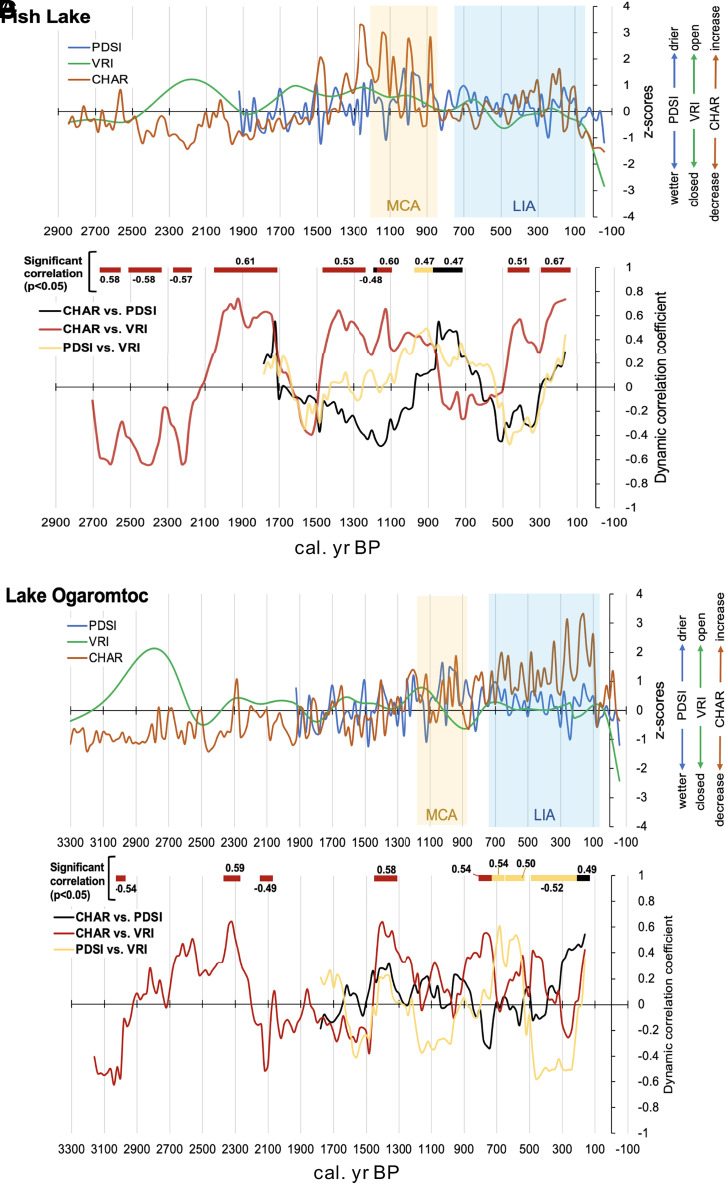
Standardized sedimentary proxies and independent climate reconstructions were interpolated at 20-y intervals using a cubic smooth spline for both lakes. VRI (green), CHAR (red), and PDSI (blue) were plotted (*A* and *C*). PDSI and VRI values were multiplied by −1 so that dry PDSI, open VRI, and increased CHAR matched direction on the axis (i.e., upward). The MCA and the LIA are shown in the yellow and blue panels, respectively. A rolling window correlation was used to estimate and plot the correlation coefficients and their respective *P* values. (*B* and *D*) Dynamic correlation coefficients were plotted for CHAR-PDSI (black), CHAR-VRI (red), and PDSI-VRI (yellow). Significant correlations are shown above the plots, with colored bars matching the color of the correlated proxies (i.e., black, red, or yellow), along with the value of the correlation.

Known climatic anomalies, such as the LIA and MCA, are useful periods to test for human modification of the fire regime (34). By zeroing in on the LIA and MCA (34), we can detect climatically anomalous fire and vegetation dynamics using the Palmer drought severity index (PDSI), charcoal accumulation (CHAR), and a vegetation response index (VRI) (23, 24). In the scenario of lightning ignitions plus Indigenous burning, more open-canopy/shade-intolerant taxa are expected to persist during periods of cooler, wetter conditions, which will be most apparent in the LIA. That is, Indigenous burning is expected to augment the fire frequency to accentuate open-canopy conditions over what would have occurred due to climate and lightning-ignited fires alone. Pollen and charcoal records from Fish Lake and Lake Ogaromtoc are inconsistent with climatically controlled patterns of forest and fire dynamics during the LIA (Fig. 4). While some lag is expected, the response times of vegetation to climate change in previous California paleoecological studies has occurred over relatively short spans (i.e., centuries) (5, 23, 24, 40). At Fish Lake during the LIA, PDSI suggests cooler and wetter conditions were accompanied by statistically significant increases in charcoal influx and forest opening (Fig. 4 *A* and *B*). At Lake Ogaromtoc during the LIA, a statistically significant negative correlation between PDSI and VRI suggests forest opening continued (Fig. 4 *C* and *D*).

### 2.4 Cross-References Show Consistency in Biomass Records.

**Fig. 5.**
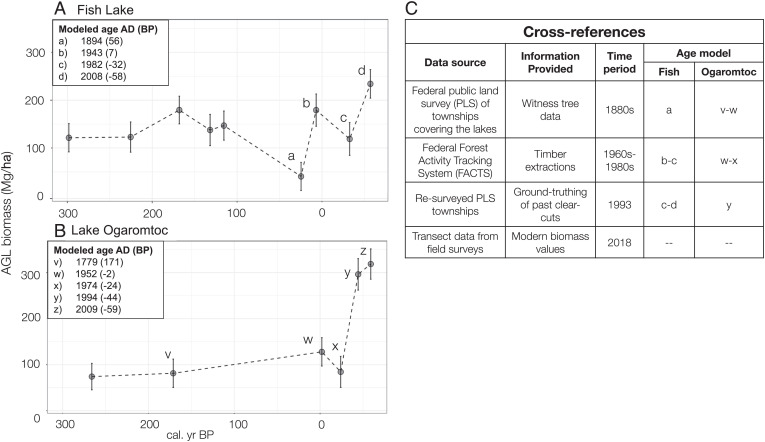
Enlargement of (*A*) the period 300 to −58 calBP with modeled ages (small letters) and corresponding predicted biomass at Fish Lake and (*B*) 300 to −59 calBP with modeled ages (small letters) and corresponding predicted biomass at Lake Ogaromtoc. Data points (green circles) with small letters were cross-referenced against independent datasets (*C*).

We used independent archival evidence to check the consistency of our biomass record from ∼150 calBP onward (Fig. 5). Predicted biomass values at both sites were generally low initially and then rose rapidly toward the present day (Fig. 5 *A* and *B*). Density calculations derived from 1880 witness tree data in the area (41) indicate low AGL biomass (mean 100 Mg/ha, SE 7.1), consistent with low predicted biomass at both lakes ∼70 calBP. We also detected reductions in pollen influx that temporally correspond to documented harvests of mature, pollen-producing trees (Fig. 5*C*). This result further confirms the link between forest biomass and pollen influx at the lake sites (*SI Appendix*, Fig. S2). Timber harvest data from the federal Forest Activity Tracking System (FACTS) database (42) indicate patch clear-cuts occurred near Fish Lake in 1968, 1977, and 1985 (*SI Appendix*, Fig. S2*A*). A corresponding drop in predicted biomass was found at the modeled age 1982 (±3 y). The FACTS database also shows patch and stand clear-cuts occurred near Lake Ogaromtoc in 1961, 1972, and 1984 (*SI Appendix*, Fig. S2*B*), the largest of which was a 105-acre (42.5 ha) clear-cut in 1972, and predicted biomass indicated a drop by 1974 (±2.8 y). US Forest Service field notes from a 1993 resurvey around the lake sites provide additional confirmation of clear-cuts recorded in FACTS, as well as a dense modern landscape (43). For example, “old clear-cut areas” are noted adjacent to Fish Lake (e.g., sections 3, 4, 9, and 10), and the general description reads: “vegetation varies from dense brush, manzanita, chinkapin, and clear-cuts” (43). Lastly, high modern biomass values in Klamath montane forests were confirmed by detailed field surveys showing modern forest biomass > 200 Mg/ha (39).

## Discussion

These results consistently show that climate alone cannot explain documented trends in forest structure and composition during the last 3,000 y. Instead, our evidence supports the premise that Indigenous land management augmented the fire regime in the western Klamath Mountains prior to Euro-American colonization. The biomass record strongly suggests frequent fire-limited biomass relative to the potential productivity of the sites. The Karuk-Yurok ethnographic data and fire scar data, supported by anomalous vegetation-fire-climate correlations during the LIA, are suggestive that Indigenous stewardship contributed substantially to the fire regime in these watersheds. Because there was similar Indigenous presence, vegetation, and climatic conditions in the low-elevation areas of the Klamath Mountains (12), these results are applicable beyond the two lake sites, indicating potentially regionwide maintenance of low forest biomass.

The most powerful way to assess the reliability of a proxy record, such as our biomass reconstruction, is to compare the reconstruction to independent archival records (26). Our biomass reconstructions are consistent with multiple, independent lines of evidence. For example, low biomass values derived from 1880 public land survey witness tree data (41) align with predicted biomass from the same period. Additionally, documented silviculture treatments since the 1960s translated into dips in the biomass record, and regrowth after clear-cut events was also captured by concomitant increases in biomass estimates. Qualitative descriptions from tribal members also link Indigenous fire stewardship practices for subsistence and open-forest conditions to the low biomass predicted by the biomass reconstruction before Euro-American colonization. The lower precontact mean biomass at Lake Ogaromtoc compared to Fish Lake (100 versus 115 Mg/ha; *SI Appendix*, Table S2) (Figs. 2 and 3) could be due to differences in soil fertility. Lake Ogaromtoc has a higher proportion of ultramafic (serpentine) substrate which tends to have lower productivity (40). However, the uncertainty in our biomass proxy method, and slightly more mesic conditions at Fish Lake due to coastal summer fog (35) that might support higher biomass, cautions against overinterpretation. Two peaks in precontact biomass were noted at Lake Ogaromtoc in 2300 and 1100 calBP (Fig. 3*A*) and are corroborated by temporally consistent peaks in *Sequoia sempervirens* pollen and heightened fog reconstructed from a nearby high-resolution paleoclimate record (44). Increased fog ∼2300 and 1100 calBP occurred due to changes in coastal currents and upwelling that may have heightened productivity or limited the spread of fires, allowing biomass to increase.

In addition to the biomass trends, the paleo–fire record matched expectations from Karuk/Yurok-based knowledge about cultural burning. Both Fish Lake and Lake Ogaromtoc had lower long-term biomass pre–Euro-American colonization, which was likely maintained by the numerous fire events detected in the charcoal influx and fire scar record. The Karuk and Yurok burned strategically and seasonally around the lakes to provide trail access and to promote desired resource qualities among their traditional gathering and hunting places (28). Cultural use of fire and Indigenous fire technology also developed, evolved, and diversified over time (27). For example, the Tuluwat Pattern (1500 calBP onward) marks a transition to more intense land usage and possibly increased tribal burning to support expanding populations driven by migration into northwestern California (24, 28). High CHAR around 1500 calBP and commensurate low biomass at Fish Lake are consistent with shifts in cultural subsistence patterns.

The relative contribution of past ignition from lightning versus cultural burns, however, is difficult to separate. In this work, 81 to 91% of fire scars were detected in latewood or at the ring boundary, similar to fire scar findings from Happy Camp, Klamath National Forest (45). Previous research has found that lightning is less likely to strike and ignite fuels in riparian areas, wetlands, prairies, and mid- to low-elevation sites, like our lake sites (32), and more likely to strike in higher elevations and ridges (46); however, lightning ignitions from distant or upper-slope fires could spread and scar trees at lower elevations months after the initial ignition. The dearth of earlywood scars could reflect the lack of lightning in the area around the lake sites, coupled with late-season cultural burning that coincided with latewood accretion, or it could reflect lightning fires that traveled from high elevation or some combination of the two. In sum, the timing of fire scar data does not conclusively support or discount accounts from traditional knowledge that Indigenous burning was the predominant source of ignition.

As expected, the lack of recent fire scars and limited charcoal influx from our record coincide with 20th-century fire suppression. Federal and state-mandated fire suppression began after the forest reserve system was established in 1905. US Forest Service and Civilian Conservation Corps suppression efforts became effective in accessible areas of the Klamath Mountains in the 1920s and in remote areas after 1945 (35). Greatly reduced fire perimeters in the California Department of Forestry and Fire Protection’s historical records demonstrate the effectiveness of this policy in Klamath and Six Rivers National Forests (41, 47). While tribal burning declined during the mid-1800s gold rush period in other portions of the Klamath Mountains (48), the evidence of fire (i.e., two or more fire scarred trees) at Lake Ogaromtoc and Fish Lake continued until 1903 and 1911, respectively. The fact that fire continued despite a reduced Indigenous population could be due to American colonists continuing to use fire to clear vegetation (35), local resistance to federal fire suppression (49), or sufficient lightning ignitions. Nonetheless, this study is broadly consistent with Klamath area fire history studies indicating two distinct fire regime periods: one before fire suppression and one after (45, 50).

If climate and climatically induced factors were the only driving forces of forest change, we would expect changes in vegetation structure and fire to be consistent with changes in local climate reconstructions. For example, a shift to drier climatic conditions (+PDSI) may elevate charcoal production (+CHAR) through increased fire frequency or higher severity fire, both of which promote forest opening (+VRI). As in other multidisciplinary paleoecological research (23, 24), we found pollen assemblage dynamics were not always well predicted by climate. We documented increases in charcoal coupled with increased shade-intolerant taxa at Fish Lake and Lake Ogaromtoc during the LIA, a climatically cooler and wetter period during which the succession to shade-tolerant species absent frequent human ignitions would be expected. We infer from the aforementioned oral histories and fire scar record that Indigenous burning was at least partly responsible for fires during the LIA. The cool, moist conditions of the LIA coincided with tribal population growth and cultural expansion, evidenced through increased trading between the coast and the interior (27). By 700 calBP, subsistence activity increased at midelevation, foothill, and valley riverside sites, suggesting larger populations and/or territoriality pressures (51). After the LIA, more fires, a more open landscape, and decreasing biomass would be expected. Instead, fire suppression and rising forest biomass driven by increasing abundance of fire-intolerant *Pseudotsuga* and *Notholithocarpus* at the expense of shade-intolerant taxa such as *Quercus* were pronounced, corroborating other records during the last century (41, 52).

Climate is undoubtedly a major factor in past and present fire regimes, but humans are also important drivers of ecosystem change and respond to climate, producing complex vegetation dynamics (53). Our biomass and fire history findings align with a growing body of literature corroborating ethnographic accounts of the influence of Indigenous land management on landscape-scale vegetation in California (12, 23, 28) and North America more broadly (54). Debate about the extent of Indigenous landscape modification, however, still exists. In California and other North American landscapes, some have argued the impact of Indigenous burning at a regional scale was negligible, and the effects of climatically driven fires exceeded anthropogenic fires (6) in the Sierra Nevada (5) and northeastern United States (55).

Discrepancies between results of paleostudies stem, in part, from differences in spatial scale. In contrast to regional perspectives (6, 55), this study relied on two small lakes which inherently reflect local vegetation and fire history (39). Additionally, the signal of Indigenous impact in the paleorecord can range from slight to ecologically profound (2). Successful detection of an Indigenous signal can be obscured by several factors. For instance, these lakes were selected in part because the surrounding landscape has supported Indigenous inhabitants and these lakes remain culturally important to local tribes. Depending on the location of sedimentary deposits relative to Indigenous presence, Indigenous management may not be captured in the sedimentary record but could still have influenced forest composition (56). Consulting local tribes who could gauge the potential detectability of their practices in the paleorecord is critical (11). For example, we participated in the Karuk Tribe’s Practicing Pikyav policy for collaborative research (57). This policy not only protects tribal intellectual property but also enriches research design.

This research underscores the need to develop a more accurate and quantitative representation of past forest biomass that takes Indigenous influence on fire regimes and vegetation dynamics into account. To restore more resilient forests, managers often rely on historic conditions as a baseline; these conditions are assumed to reflect a natural range of variation in the absence of human modification (58). By assuming historical forests were not highly managed, the long-term role of Indigenous people is discounted. A major drawback of this assumption is it understates the scale of intervention needed to achieve historical fidelity. Our work, in contrast, suggests Indigenous forest and fire management played an important role in maintaining forest conditions before Euro-American colonization. The study also illustrates the unprecedented level of contemporary forest biomass and puts the last century of fire suppression into its long-term context. Predictable dynamics of forest carbon storage are needed to achieve California’s greenhouse gas emissions goals (59), yet the contemporary biomass record is unstable in comparison to the long-term trend (Figs. 2 and 3). Indigenous knowledge and cultural practices are rarely translated into land management plans despite their value (11). This work demonstrates the power of integrating paleo- and ethnographic records to inform land management geared toward historical fidelity.

## Materials and Methods

### Karuk/Yurok-Based Indigenous Knowledge.

We report ethnographic information from archived interviews conducted with Karuk and Yurok tribal members (12, 30), as well as historical documents (60) and recent surveys of tribal members and associated cultural burning practices (38). *SI Appendix* contains all relevant materials. Full quotes from *section 2.1* are presented here.

“Fire is crucial to who people are, and what they do. It enables them to live. It is a central component of that duty of care for the whole world, which is inherited from their common ancestry as Spirit People” (30).

Fish Lake and Lake Ogaromtoc (Frog Pond), as described by Karuk tribal member, Charlie Thom in 1996 (31):

“And there was an abundance of stuff. My family gathered at [nearby places]…and our gathering place for acorns at Frog pond [Lake Ogaromtoc]. Beautiful, beautiful acorn gathering place and mushroom gathering place.”

Fish Lake was historically used as a gathering place by the Yurok (60):

“Our first sleep…would be spent by the borders of a small lake to the north of Weitchpec, among the pine and fir timber. After we had followed a trail a mile or more up the river, we began to ascend the mountain…The lake, but few acres in extent, and almost covered with pond-lily pads, contained an abundance of trout, upon which we feasted.”

The Karuk and Yurok have described modern forest conditions (38):

“…and degradation of the environment are reported as the strongest barriers to accessing native foods…”

### Paleoecological Records.

#### a. Predicted AGL tree biomass.

PAR (pollen accumulation rate) values were calculated using

[1] $$PAR_{i}=C_{i}\times S,$$

where *PAR_i_* is the PAR value for taxon *i*, *C_i_* is the pollen concentration (in grains per cubic centimeter) for taxon *i*, and *S* is the sedimentation rate (in centimeters per year) which was determined above in *section 2.2* (61). Knight et al. demonstrated that PAR values of major tree taxa derived from lake sediments are linearly related to distance-weighted AGL biomass (39). Biomass trends were plotted using palyoplot (62) in R (63). Error in the biomass estimate based on PAR measurements was propagated using a resampling method (64). Specifically, we estimated error in the predicted AGL biomass as a random sample from a normal distribution with the mean equal to zero and the SD equal to the SE of the regression estimate for each taxon. For each iteration, we included a taxa-specific live biomass estimate based on its PAR value and summed results for each core sample (i.e., lake and time specific) to estimate the AGL biomass. Uncertainty was calculated from 10,000 iterations and reported as means and SEs of the predicted AGL biomass for each sample.

#### b. Fire scars.

Cross-sections of stumps/downed logs with visible fire scars were collected around the lakes in 2008 to 2010. Each cross-section was sanded, allowing tree rings and fire scars to be distinguished under a microscope, and then cross-dated against tree-ring chronologies from nearby locations (65, 66) using standard dendrochronological methods (67). The COFECHA program was used to identify most likely ring dates for samples difficult to cross-date (68). Fire scar dates were plotted using the Fire History Analysis and Exploration System (69). To reduce the chance of including scars caused by very small (i.e., single tree) fires or wounding other than by fire, a composite of fire years was generated using only scars recorded by a minimum of two trees per site. The median fire return interval for each site was calculated for the period from 1700 and 1900, the time frame during which sample depth was maximized.

### Correlations among Climate, Vegetation, and Fire Proxies.

#### a. Independent climate analysis.

Independent, annually resolved climate reconstructions for the Klamath bioregion over the last 2,000 y come from the North American Drought Atlas (NADA) tree-ring datasets (70). Tree-ring reconstructions were used to calculate annual paleo–drought conditions based on the PDSI. Annually reconstructed PDSI values from grid cell 035 of the NADA were used as an independent measure of climate change at both lakes.

#### b. Vegetation response index.

Changes in the percentage of pollen taxa over time are used to interpret changes in surrounding vegetation (71). When taxa respond inversely to climatic variability, a single-variable VRI can be calculated from the ratio of different taxa to clearly illustrate change (71, 72). A shade tolerance scale of Northern Hemisphere trees and site-specific knowledge was used to determine which taxa to compare (73, *SI Appendix*, Table S1). The VRI was calculated from pollen counts: ((*Pseudotsuga* + *Notholithocarpus*) − (*Quercus + Pinus*))/(*Pseudotsuga* + *Notholithocarpus* + *Quercus + Pinus*). Positive VRI indicated a greater proportion of shade-tolerant to shade-intolerant pollen and was inferred to show a more closed canopy, while a negative VRI indicated a greater proportion of shade-intolerant to shade-tolerant pollen and was inferred to show a more open canopy. Results from a nonmetric multidimensional ordination of tree abundance (as measured by the AGL biomass) at different sample dates supported the interpretation of the VRI (74, *SI Appendix*, Fig. S4).

#### c. Charcoal influx data.

Continuous 1-cm^3^ samples of macroscopic charcoal were collected in each core, corresponding to a time resolution of ∼3 y. Macroscopic charcoal preparation steps are detailed in Crawford et al. (24) and followed standard procedures (75). Charcoal influx data provide a qualitative reconstruction of fire activity (76). Char-Analysis charcoal peak methodology and results are described in *SI Appendix*, Fig. S5 *A* and *B* (77).

We interpolated PDSI, VRI, and charcoal influx every 20 y using a cubic smooth spline (Fig. 4). We estimated correlation coefficients and their respective *P* values using a rolling window approach with 20-y time steps (78) with the R package RolWinMulCor (78). This package relies on the Benjamini and Hochberg method to correct *P* values for multiple comparison and autocorrelation.

### Cross-References.

In QGIS version 3.14 (79), lake boundaries (80) and harvest records (42) were obtained and plotted. Federal public land surveys from 1882 and 1993 were obtained from the Bureau of Land Management (41). Detailed transect surveys between 0 and 750 m from the lakes’ shores were undertaken in 2018 (39).

### Site Description.

This study presents data from two small lakes with small watersheds and minimal stream inputs from the western Klamath Mountains (Fig. 1). We reanalyzed records from previously collected (2008 to 2009) sediment cores at Fish Lake and Lake Ogaromtoc (24). Vegetation at both sites is composed of montane hardwood-conifer forests with a diverse, well-mixed canopy of tree species (*SI Appendix*, Table S1) (24). The climate of the Klamath region is best described as Mediterranean with cool, wet winters and warm, dry summers (35). Before 20th-century fire suppression, the landscape had a mixed-severity fire regime characterized by frequent, mostly small, and low-intensity fires and less frequent (fire rotations of ∼15 to 30 y), large, and mixed-severity fires (35). Due to mountainous topography, fires burned with great spatial complexity, creating openings of variable sizes (35). Locally, Native burning and selective encouragement of species had significant effects on vegetation structure and composition (24).

### Chronology and Laboratory Analyses.

Details about sediment coring, ^14^C dating, and ^210^Pb dating are previously published (24). Using raw ^210^Pb activity data and uncalibrated ^14^C data (81), we constructed age models for Fish Lake and Lake Ogaromtoc using the Bayesian-based software Plum (82). The rplum package (83) integrates lead and carbon together (*SI Appendix*, Fig. S3). Deposition times (in years per centimeter) were quantified at every section where pollen was sampled and converted to accumulation rates (in centimeters per year) for PAR calculations. Pollen samples were extracted from 0.625 cm^3^ of wet sediment at increments of ∼5 to 10 cm down each core (corresponding to a time resolution of ∼15 to 30 y for VRI) and completed previously (81). Additional samples were extracted from the Lake Ogaromtoc core to refine existing pollen data, but the Fish Lake core was not suitable for resampling. All samples were processed using the same standard methods (84). *Lycopodium* spore tracer tablets were added to determine pollen concentrations (85).

## Supplementary Material

Supplementary File

## Data Availability

Pollen count and fire scar data have been deposited in Neotoma (https://data.neotomadb.org/20495; https://data.neotomadb.org/20316).
