# Supplementary material for: Land management explains major trends in forest structure and composition over the last millennium in California’s Klamath Mountains
Source: Proc Natl Acad Sci U S A. 2022 Mar 14;119(12):e2116264119. doi: 10.1073/pnas.2116264119 (PMC8944927; doi:10.1073/pnas.2116264119)
Supplement: Supplementary File [file pnas.2116264119.sapp.pdf]

Supplemental Information

Title: Land management explains major trends in forest structure and composition over the last millennium in California's Klamath Mountains

Authors: Clarke A. Knight[1,2], Lysanna Anderson[1], M. Jane Bunting[3], Marie Champagne[1], Rosie M. Clayburn[4], Jeffrey N. Crawford[5], Anna Klimaszewski-Patterson[6], Eric E. Knapp[7], Frank K. Lake[8], Scott A. Mensing[9], David Wahl[1,10], James Wanket[6], Alex Watts-Tobin[11], Matthew D. Potts[2], John J. Battles[2]

[1] U.S. Geological Survey, Menlo Park, CA 94025 USA

[2] Department of Environmental Science, Policy, and Management, University of California, Berkeley, Berkeley, CA 94720 USA

[3] Department of Geography, Geology and Environment, University of Hull, Cottingham Road, Hull, HU6 7RX UK

[4] The Yurok Tribe's Cultural Resources Manager, Klamath, CA 95548 USA

[5] USDA Forest Service, Deschutes National Forest, Bend, OR 97701 USA

[6] Department of Geography, California State University Sacramento, Sacramento, CA 95819 USA

[7] US Forest Service Pacific Southwest Research Station, Redding, CA 96002 USA

[8] US Forest Service Pacific Southwest Research Station, Arcata, CA 95521 USA

[9] Department of Geography, University of Nevada, Reno, Reno, NV 89557 USA

[10] Department of Geography, University of California, Berkeley, Berkeley, CA 94720 USA

[11] The Karuk Tribe's Department of Natural Resources, Orleans, CA 95556 USA

Corresponding author: cknight@usgs.gov

Note: Any use of trade, firm, or product names is for descriptive purposes only and does not imply endorsement by the U.S. Government.

In addition to results presented in section 2.1, we present other relevant ethnographic and historical literature about the western Klamath Mountains (A), as well as other lines of evidence from Euro-American qualitative descriptions (B).

A. Documentation of tribal uses of Lake Ogaromtoc and Fish Lake

From Karuk Tribe Department of Natural Resources (1999, Vol 1):

1. Gathering Areas (pp. 57):
   "And there was an abundance of stuff. My family gathered at [nearby places]…and our gathering place for acorns at Frog pond [Lake Ogaromtoc]. Beautiful, beautiful acorn gathering place and mushroom gathering place. You got to stop burning Frog Pond. Like I said, they try to burn everything down and they don't realize there's a cloud coming in from the coast, coming in, let her hang up a little while there. Acorns, tan oak will hold back the moisture… I see so much clear cuts on the Klamath River now and doing more. I was totally against them guys clear cutting Frog Pond. Beautiful, beautiful acorn gathering place and mushroom gathering place. A lot of basket materials. All of that stuff was there. Then to top it off, not only did they wreck a Medicine Trail, sacred Indian Trail, bull dozers and loggers …just ruined an Indian trail. The sacredness of that trail, very important to Mother Nature. Mother Nature itself. Cause you don't just walk down a sacred trail cause it's a trail. The sacredness of the trails going somewhere, leading you to somewhere, a special place in northern California mountains, on the Klamath National Forest. Destroyed. 'Where's that trail? It used to once be right there.' No, and they destroyed it." Charlie Thom, interview conducted 11/96.

From Karuk Tribe Department of Natural Resources (1999, Vol 2):

2. Basket Materials/Gathering Issues (pp. 2):
   "…then sometimes they'd [Indians] walk for miles to gather their materials. You'd try to find it close to your residence[village/homestead-allotment]. And they used to go over Rock Creek, over that way. And there used to be a fire burning area there for the bear grass. They traded things like spruce roots with the other side [coast Yurok/Tolowa].

They'd trade acorns and things that we had that were from here for them. Over there we'd get shells and whatever. So, it was a good way to go." Vera Arwood, interview conducted 08/07/1997.

3. Fire (pp. 42):
"My dad lived five miles up Bluff Creek at the Garnet Ranch. In the fall of the year, it would get so smoky that you couldn't even see. It never burned nothing." Page 43: "This brush situation is getting damn tough around this place. There should be something done about it. They'd burned up here last year and then at Frog Pond. It got pretty hot in some places. They need to cut the stuff down so it will be on the ground and won't burn up into the trees. They need to burn up some of these piss ants and other bugs." Ernie Spinks, interview conducted 11/1996.

Oral histories/informal interviews:

4. Norman Goodwin, Karuk Ceremonial Leader/elder. Informal discussion with Frank K. Lake at the Happy Camp-Ishkeesh Brush Dance, July 31, 2010. Norman said that there was an "old [Indian] trail that followed the serpentine ridge above Frog pond along the divide of Bark Shanty Gulch (tributary to Rock Creek) that head around/above Frog pond on the west/southwest to south/south east side towards the Klamath River. The trail connected from Eyese Village along the west side of the Klamath River.

5. Charlie Thom, Karuk Ceremonial Leader/elder. Informal discussion with Frank K. Lake at Katamin/Somes Bar "War Dance/gathering" May 29, 2010. Charlie said that Frog Pond was used as a camp for Indian Doctors. Coming from nearby villages on their way up to and back from the Elk/Flint Valley "High Country" [Southern Siskiyou Wilderness] area.

6. Kathy McCovey-Barger, Karuk Brush dance medicine woman/USFS Archaeologist. Informal discussion(s) with Frank K. Lake during a district archaeological/cultural resource tour on August 6, 2010. Kathy stated that she was told from Karuk elders that

Frog Pond may have been a "village" at one time and asked if I found the "house pits" or other features.

7. Other related cultural or archaeological information. Frank Lake observations at Frog Pond (Lake Ogaromtoc):
    a. Siskiyou Wildfire 2008. South/south east side of pond, along flat. Found old Indian cooking fire-cracked rocks.
    b. Fire history sample collections Aug. 2010. Found granite stone hand grinder along serpentine ridge on west side of pond. Also, white quartz flake fragments (non-native soil at that place, later found natural white quartz rocks to the southeast, up the ridge about ½ from fragments).

Other relevant publications:

8. Yurok story of mother and son. Taking the "inland" trail back from Orleans (Karuk: Panamnick village") to her home. This trail went to and past Fish Lake over Kewet Mountain = Burrill Peak [Rivet Mt. circa 1920s maps] to down river Yurok village: Pages 24-25 visits to Fish lake: "…the two of them wandered on up through the hills as far as a small mountain lake called Fish Lake…made camp. They slept there the rest of the night by the lake; Son (*Toan*) going hunting/trapping Pages 28-29: "As *Toan* grew older and went hunting along or with other boys, he continued to bring his great-grandfather little birds, then bigger birds, and especially the red-headed woodpecker, and then the fur animals-whatever he could hunt or trap…*Toan* sometimes hunted inland on *Kewet* Mountain and as far as Fish Lake. Whenever he was at the lake, he wondered what had become of the log on which he had climbed that first time…"; Author's discussion narrative Pages: 165-166 "The journey undertaken by the young mother of the story is accomplished as such a journey on foot would be today (see map accompanying the story): by trail from *Ko'otep*, skirting the river to the spot on Camp Creek (downriver side of Orleans, Karuk *Tishunik*, village and ceremonial grounds) where the Deerskin Dance was held. The return follows the same trail as far as Red Cap Creek; then inland away from the river, passing along Fish Lake and by *Kewet* {Burril Peak} Mountain, thus

bypassing the populous village of *Weitspus*, coming out on the river again at *Murek*, and from there downstream to *Ko'otep* once more. (Kroeber 1959)

9. "A small circular lake. In myth times a number of 'people' gathered at this point to shoot a gigantic bird [condor] with wonderful feathers, which occupied the summit of the mountain *hegwono'L* (Shelton's butte), lying to the southeast, across the Klamath". (Waterman 1920) *See Rectangle G, Map 25. Notes on page 256: Site 1. "itprpr'".

10. "Our first sleep…would be spent by the boarders of a small lake to the north of Weitchpec, among the pine and fir timber. After we had followed a trail a mile or more up the river, we began to ascend the mountain…The lake, but few acres in extent, and almost covered with pond-lily pads, contained an abundance of trout, upon which we feasted" (Pearsall and Pearsall 1928).

11. The best tanoak tree orchard acorn areas are fully grown/crown structure trees that are burned about 3-5 years to reduce acorn pests (weevil/moth) and enhance gathering by having a low fuel ground surface. Craig Erwin, a Yurok man, noted that "They [the Yurok] burned the hazel sticks and all the basketry materials, very important, and also important they burned for the wildlife, the deer and the animals that needed the burn. ...They also need to burn because the oak trees will become what they call wormy. They'll get a worm in the acorns, and the Yurok word is *tirpir* [phonetic spelling]—it's a little worm that gets in the acorns and destroys the crops. So by burning, that's how they kept disease from the forest..." (Riley-Thron 2001).

B. Additional lines of evidence, including Euro-American qualitative descriptions:

12. Chartkoff and Chartkoff (1975):
"The settlement data provided by Kroeber and Bright allowed some observations to be made as to locational variability among the Karok. Bright's map (1957) showed that settlements were most densely clustered around the confluence of the Klamath and Salmon, and were the sparsest in the northern half of the Karok territory, above Clear

creek. Kroeber's data (1936) listed the reported number of households for most of the more than 100 Karok settlements he described; they ranged from one to ten, with a mean of from six to seven. The average number of persons per household was 7.5 (Kroeber 1925). The Karok lived along 100 km of the Klamath and 20 km of the Salmon, with a population estimated at 2,700 (Baumhoff 1963). Those figures give an average of 22.5 persons per kilometer of river. With those data it was possible to predict densities of people along the river at different points."

13. Nineteenth century descriptive evidence from Euro-Americans provides additional corroborating insight into the landscape. The federal public land survey (PLS) conducted in 1882 at the townships covering Fish Lake and Lake Ogaromtoc documents that the area around Fish Lake contained a "number of settlements", "prairie and grassland", as well as "large bodies of fine sugar pine timber of good quality" (Foreman 1882a, 10N4E). At Lake Ogaromtoc, PLS surveyors witnessed a landscape "covered with pine and oak and considerable sugar pine of good quality" (Foreman 1882b, 13N6E). For the Karuk and Yurok, *Pinus lambertiana* (sugar pine) wood was a prized fire starter, and *P. lambertiana* stands were cultivated and managed by tribal groups for pinenuts (Schenk and Gifford 1952).

14. A traveler's 1908 account described considerable oak populations in the Bluff Creek area near Fish Lake: "the hills were covered with white oak and madrone… the rocky areas were covered with live oaks" and "a few sturdy firs scattered among the oaks" (Baldwin 1990).

15. Until the 1920s, one of the main passageways from Karuk habitation on Rock Creek southwards was along the ridgeline where Lake Ogaromtoc is situated. Based on the tribal lore and experiences of the Yurok and Tolowa, Warburton and Endert (1966) report that: "It was common sight in the 1880s to see five or ten Indian men and women heading for their homes with heavy burdens of food, over almost obscure trails…having come ten to fifteen miles in one day over steep trails". Many of these trails became USFS roads that facilitated timber extraction and fire suppression in the 1950s.

16. Although we expected tree biomass to increase and coincide with land use change starting in 1850, the magnitude of the shift was large. For instance, the predicted biomass at Lake Ogaromtoc in 1950 was 126 Mg/ha and rose to 320 Mg/ha by 2008. This increase in biomass in only a few decades is plausible: a 100 Mg/ha *P. menziesii* dominated forest grew to 360 Mg/ha in 40 years (Stewart and Sharma 2015). As a result of recent rapid increases in forest biomass, modern forest structure is unprecedented in the last 3,000 years.

Additional results supporting the distinction of vegetation types

The two most abundant species are *Pseudotsuga menziesii* and *Notholithocarpus densiflorus,* with *Pseudotsuga menziesii* currently more abundant at Fish Lake and *Notholithocarpus densiflorus* more abundant at Lake Ogaromtoc. Mature overstory vegetation at both sites also includes: *Chamaecyparis lawsoniana, Pinus lambertiana, Arbutus menziesii, Alnus rhombifolia, Chrysolepis chrysophylla, Calocedrus decurrens, Quercus kelloggii,* and *Quercus garryana* (nomenclature follows Hickman 1993).

We used ordination analysis to understand the vegetation groupings over time. Results support the vegetation response index (VRI) separation (*Quercus*/*Pinus* versus *Pseudotsuga*/*Notholithocarpus*) based on the shade tolerance scalar (Table S1). Although these groups occupy separate regions of ordination space and they divide along gradients in PDSI and CHAR, there are not strong gradients in the data. This is not surprising given that this study leverages two nearby lakes and analyses are limited to a time of vegetation stability.

Table S1 A classification of major pollen types by shade tolerance using a scale of Northern Hemisphere trees (Niinemets and Valladares 2006); the tolerance scale ranges from 0 (no tolerance) to 5 (maximal tolerance) with standard error estimates (SEE). Pollen types are organized from least tolerant (*Alnus*) to most tolerant (*Abies*).

**Pollen type**	**Contributing species**	**Shade tolerance scale**
Alnus	*Alnus rhombifolia*	1.5
Quercus	*Quercus kelloggii*	1.55
	Quercus garryana	2.4±0.3
Total *Pinus*	*Pinus ponderosa*	1.64±0.15
	Pinus jeffreyi	1.74±0.26
	Pinus lambertiana	2.66±0.14
Pseudotsuga	*Pseudotsuga menziesii*	2.78±0.18
Notholithocarpus	*Notholithocarpus densiflorus*	3.67±0.33
	Chrysolepis chrysophylla	3
TC	*Calocedrus decurrens*	3.21±0.53
	Chamaecyparis lawsoniana	3.67±0.38
Abies	*Abies magnifica*	3.5±0.22
	Abies concolor	4.33±0.28

Estimating forest biomass prior to European-American contact

To estimate the central tendency in the aboveground live tree biomass (AGL) at our sites, we fit a locally weighted smoothing regression (loess) to the AGL estimates at each lake (Fig. S1A,B). Given the changes in the pollen deposition rate and our systematic pollen sampling of the lake core (i.e., every 10 cm), the observations are not evenly distributed in time. In addition, at Lake Ogaromtoc, we intensified our sampling to refine the timing and magnitude of two peaks occurring ~2300 calBP and ~1100 calBP (the Fish Lake core was not suitable for additional sampling). The smoothing regression provides a means to normalize sample intensity and avoid over-representing any time period. We limited the regression to data prior to 100 calBP (1850). We generated predicted AGL by date at 50-year intervals (Table S2). The baseline AGL pre-contact was defined as the median of these predicted values. During the last three millennia, both sites maintained relatively low forest biomass values with median values between 128 Mg/ha at Fish Lake and 104 Mg/ha at Lake Ogaromtoc.

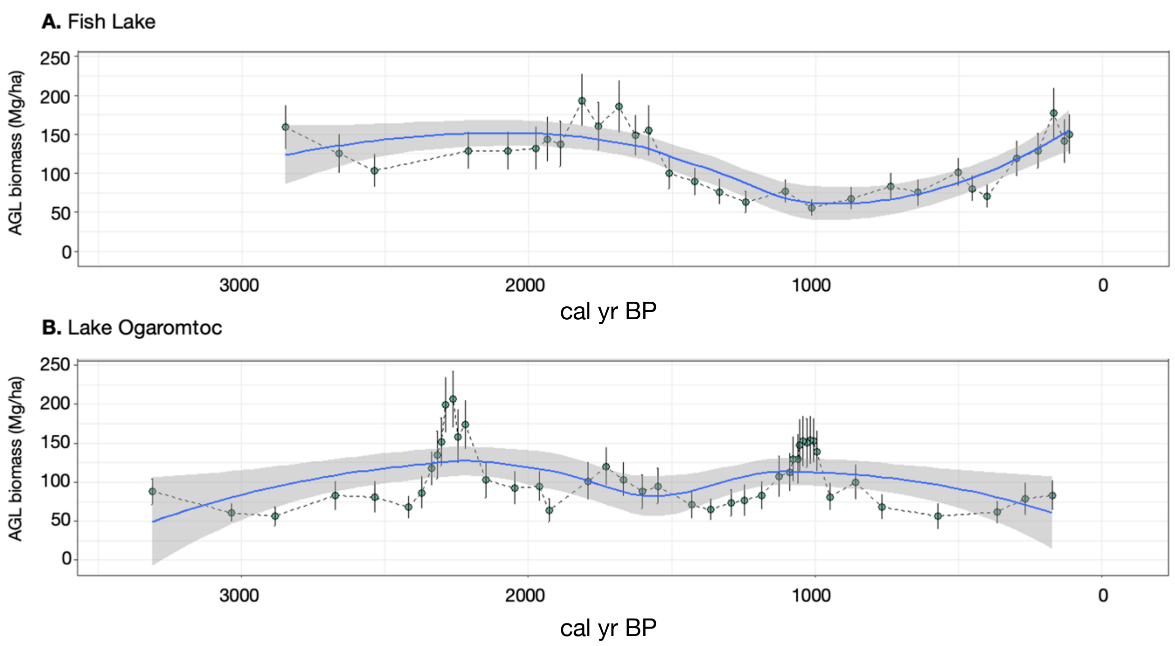

Figure S1. Trends in estimated aboveground live tree biomass (AGL) at **(A)** Fish Lake and **(B)** Lake Ogaromtoc. The circles represent estimated AGL from pollen counts; the blue line represents the locally weighted regression line; and the grey band is the 95% confidence interval around the regression line.

Table S2. Summary of the locally weighted regression of aboveground live tree biomass (AGL) for the two lakes prior to European-American settlement. Standard error (se) refers to the residual standard error of the regression; n is the number of predicted AGL values at 50-year intervals.

Lake	cal year BP	mean	se	n	median	1st quartile	3rd quartile
		Mg/ha	Mg/ha		Mg/ha	Mg/ha	Mg/ha
Fish	2800-100	115	24.2	54	128	82.8	144.5
Ogaromtoc	3300-150	100	33.5	63	104	87.0	113.0

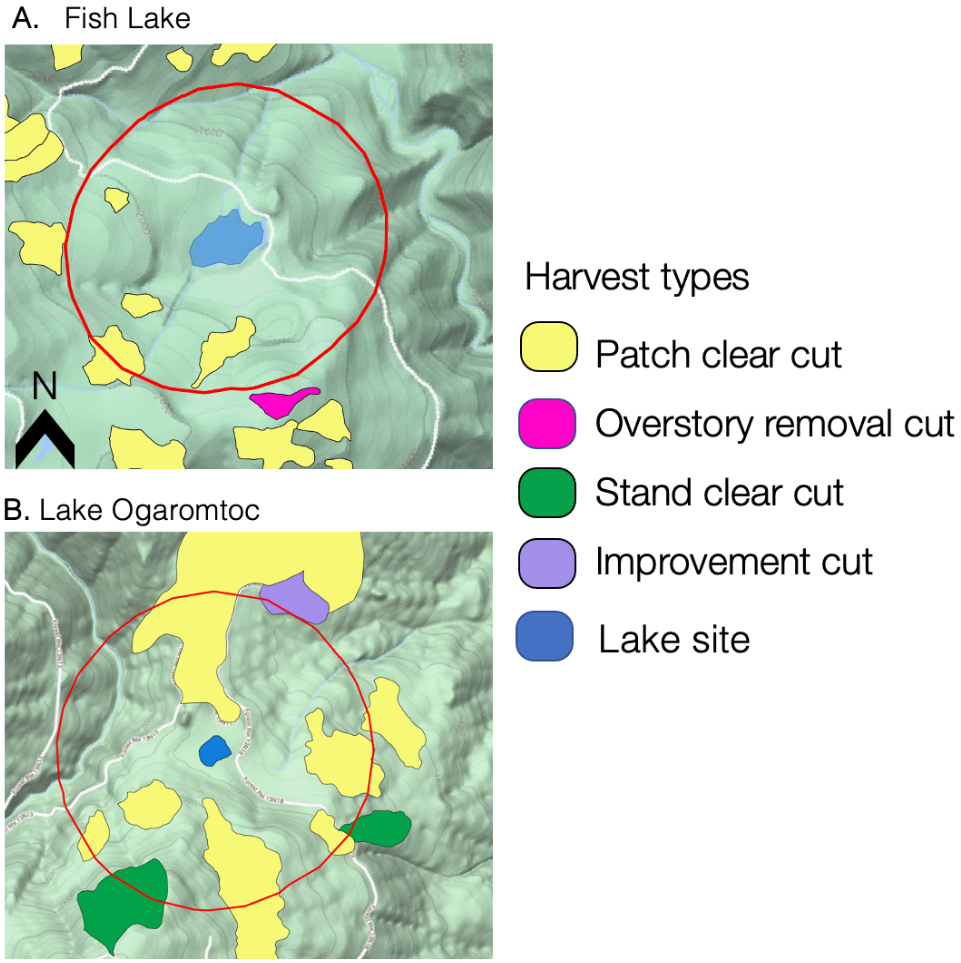

Fig. S2 Harvest perimeters from the federal Forest Activity Tracking System at **(A)** Fish Lake that took place in 1968, 1977, and 1985 and **(B)** Lake Ogaromtoc that took place in 1961, 1972 and 1984. The biomass record reflects local vegetation and potential disturbances within a ~650 m radius from the lake shore (133 ha) (Knight et al. 2021). Thin red circles demarcate the spatial extent recorded by the sediment-derived pollen record. Harvest types include: 1) patch clear cuts which are a type of stand clearcutting where patches (or strips) are clear cut within an individual stand boundary in two or more entries to produce an even-aged stand (yellow); 2) overstory removal cuts which are a final removal cut that releases established regeneration from competition with the overwood after it is no longer needed for shelter under the shelterwood regeneration method (pink); 3) stand clear cut which is an even-aged regeneration or harvest method that removes all trees in the stand producing a fully exposed microclimate for the development of a new age class in one entry (green); 4) improvement cut which is an intermediate treatment made in a stand, pole-sized or larger, primarily to improve composition and quality by removing less desirable trees of any species (purple).

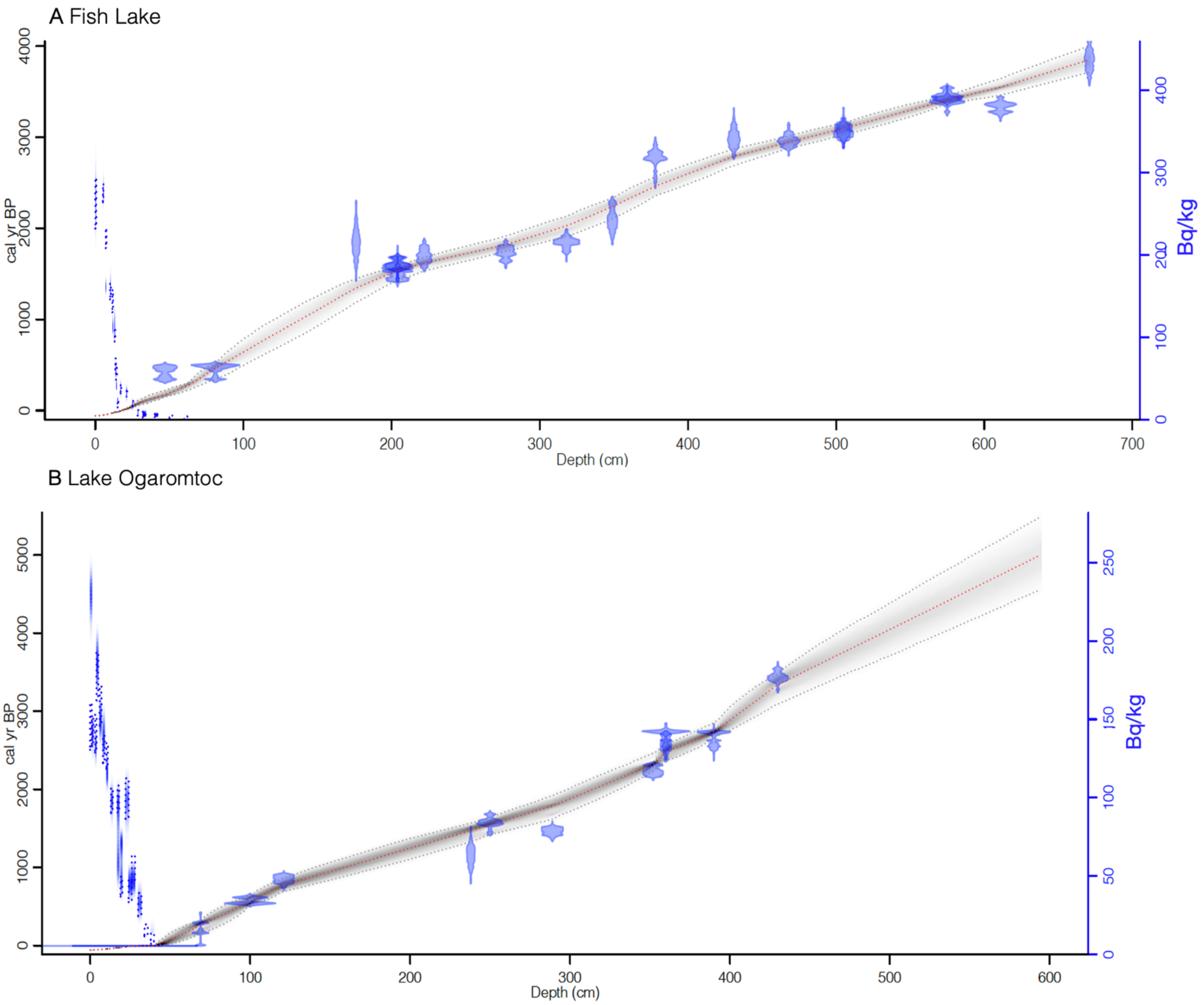

Fig. S3 Age-to-depth models for **(A)** Fish Lake and **(B)** Lake Ogaromtoc using Plum software (Aquino-López et al. 2018, Blaauw et al. 2020), which incorporates both ^{210}Pb dates and ^{14}C dates. Data from Crawford et al. (2015). The 2σ uncertainty band around the age model is enclosed by dotted grey lines; the highest probability age is the darkest grey shading and the red line represents the mean age. The second y-axis (blue, right) shows the ^{210}Pb activity in Bq/kg.

Vegetation Response Index (VRI)

For our study sites, we established a gradient in shade tolerance among the common tree species. We included *Pseudotsuga* and *Notholithocarpus* to represent shade-tolerant taxa and *Quercus* and *Pinus* as shade intolerant taxa (Niinemets and Valladares 2006, Table S1). The VRI was calculated from pollen counts: ((*Pseudotsuga* + *Notholithocarpus*) – (*Quercus* + *Pinus*))/(*Pseudotsuga* + *Notholithocarpus* + *Quercus* + *Pinus*). Positive VRI indicated a greater proportion of shade tolerant to shade intolerant pollen. Increases in VRI were interpreted as transitions toward more closed canopy forests. A negative VRI indicated a greater proportion of shade intolerant to shade tolerant pollen. Decreases in VRI were interpreted as transitions toward a more open canopy. Results from a non-metric multidimensional ordination (NMS) of tree abundance (as measured by AGL) at different sample dates supported the definition and interpretation of the VRI. In the 2-dimensional solution with the lowest stress (stress = 13.5), *Pseudotsuga* and *Notholithocarpus* clustered together in the upper left quadrant (Fig. S4). *Quercus* occurred on the edge of the diagonally opposite quadrant and *Pinus* was located closer to *Quercus* than either *Pseudotsuga* or *Notholithocarpus.* In addition, the ordination gradient from *Pseudotsuga* to *Quercus* was positively correlated with charcoal accumulation. However, the relatively high stress suggests that while the NMS provides a useful overview of the gradient space, the details of the analysis are less reliable (McCune et al. 2002).

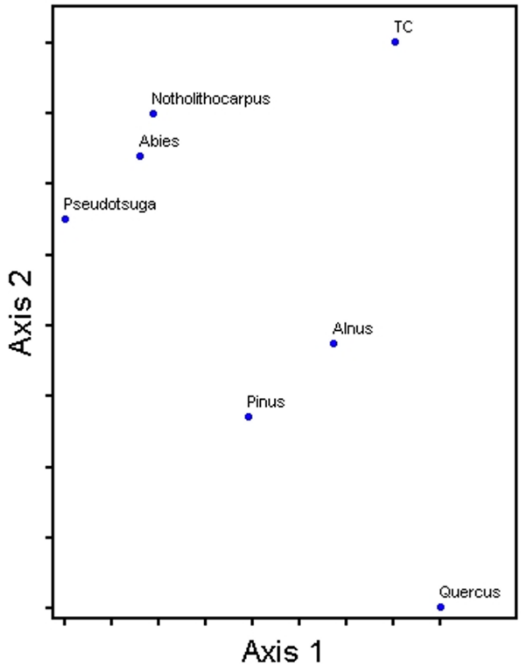

Figure S4. Two-dimensional solution of a non-metric multidimensional (NMS) ordination of forest composition at Fish Lake and Lake Ogaromtoc. Samples based on reconstructions of taxon-specific aboveground live biomass from pollen samples across a 3000-year gradient. NMS was calculated using PC-ORD v7 software (McCune et al. 2002) using a thorough search for the best solution (e.g., 400 iterations, 50 randomized runs). Stress of two-dimensional solution = 13.5.

Charcoal peaks using Char-Analysis

Significant charcoal peaks were determined using Char-Analysis software (Higuera et al. 2009). The minimum background smoothing was determined by dividing the record length (in years) by the number of charcoal samples and multiplying by 30 and "significant" peaks were those with a signal-to-noise index above 3.0 (Higuera et al. 2009). Significant charcoal peak records for Fish Lake (n=8) and Lake Ogaromtoc (n=5) (Fig. S5A,B) underestimated fire events when compared to the fire scar record, which is already known to be a conservative estimate of paleo fire. Nonetheless, we found time periods where charcoal peaks were consistent with biomass. At Fish Lake between 1500–650 calBP, biomass dropped and remained under 100 Mg/ha, coincident with significant charcoal peaks between 1500–900 calBP (Fig. S5A). At Lake Ogaromtoc, large charcoal peaks were associated with co-occurring reductions in biomass (Fig. S5B); for example, charcoal peaks at ~3,400 calBP and ~2300 calBP coincide with decreasing biomass. Biomass fluctuated at Lake Ogaromtoc but generally hovered around 100 Mg/ha between 2200 to 1200 calBP, during which time charcoal influx gradually increases. An absence of significant charcoal peaks between 1200 and 1000 calBP co-occurred with rising biomass which peaks at 1050 calBP to ~150 Mg/ha.

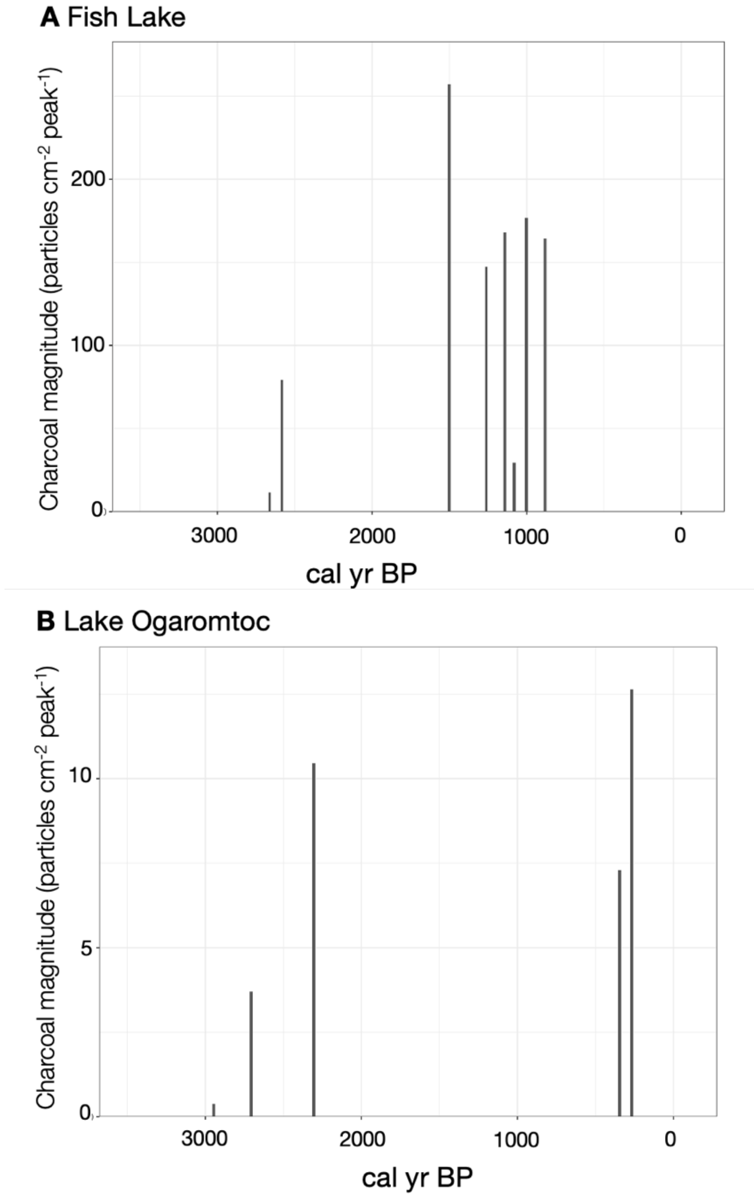

Fig. S5 Charcoal peaks were determined for **(A)** Fish Lake and **(B)** Lake Ogaromtoc using Char-Analysis software (Higuera et al. 2009). Charcoal peak (C_{peak}) was calculated as $C_{peak} = C_{interpolated}$ – local threshold value, where the peak significance threshold was set to the 95th percentile, signal-to-noise index >3, and background smoothing set to 200 years.

Rolling Window Correlation Analysis

We analyzed the correlations among charcoal peaks (CHAR), vegetation response index (VRI), and Palmer Drought Severity Index (PDSI) for the entire record using a rolling window approach (Polanco-Martinez 2020). This technique allowed us to estimate bi-variate correlation coefficients and their respective p-values without relying on a particular functional form. The downside is that the analysis makes multiple tests as it steps through the rolling window comparisons. Because testing a large number of hypotheses will produce Type 1 errors and false discoveries, we applied a correction to p-values using the Benjamini and Hochberg method (BH method). Polanco-Martinez (2020) found that the BH method was as robust as Telford (2013)'s non-parametric method that used Monte Carlo simulations to account for both multiple comparisons and autocorrelated data, but the BH method was less computationally costly. We defined our window as 380 years (19 observations of 20-year time intervals). Correlations (Pearson Product Moment) were estimated at the midpoint of each window. This size was chosen to account for the relative sparseness of pollen data points from the oldest sediments in our records. While we interpolated the VRI at every 20 years, observations in the earliest reconstructions can span more than 200 years.

Supplemental Information References

Aquino-López MA, Blaauw J, Christen A, and Sanderson NK (2018) Bayesian Analysis of 210-Pb Dating. *Journal of Agricultural, Biological, and Environmental Studies* 23:317-333.

Baldwin WC (1990) A Fish Lake Odyssey. *Humboldt Historian* 38(6):14-20.

Blaauw M, Christen JA, Aquino-Lopez MA, Esquivel-Vazquez J, Gonzalez OM, Belding T, Theiler J, Gough B, Karney C (2020) Package ‘rplum’: Bayesian age-depth modeling of ^{210}Pb-dated cores. Version 0.1.4. CRAN Repository.

Chartfkoff JL, Chartkoff KK (1975) Late period settlement of the middle Klamath River of northwest California. American Antiquity 40(2):172–179.

Cook ER, Seager R, Heim RR, Vose RS, Herweijer C, Woodhouse C (2010) Megadroughts in North America: Placing IPCC projections of hydroclimatic change in a long-term paleoclimate context. Journal of Quaternary Science 25(1):48-61.

Department of Natural Resources, Karuk Tribe (1999) Karuk Forest Management Perspectives: Interviews with Tribal Members. Vol. 1: Report and Interview Transcripts.

Department of Natural Resources, Karuk Tribe (1999) Karuk Forest Management Perspectives: Interviews with Tribal Members, Vol. 2: Appendices.

Foreman SW (1882a) Field notes for the subdivision lines of township 10 north range 4 east Humboldt Meridian, California. Digital archive from the Cadastral Survey Office, Bureau of Land Management Survey Records, Sacramento, California. Volume 323:233-299.

Foreman SW (1882b) Field notes for the subdivision lines of township 13 north range 6 east Humboldt Meridian, California. Digital archive from the Cadastral Survey Office, Bureau of Land Management Survey Records, Sacramento, California. Volume 41:1-67.

Hickman JC (1993) The Jepson manual: higher plants of California. University of California Press, Berkeley, California, USA.

Higuera PE, Brubaker LB, Anderson PM et al. (2009) Vegetation mediated the impacts of postglacial climatic change on fire regimes in the south-central Brooks Range, Alaska. *Ecological Monographs* 79: 201-219.

Knight CA, Baskaran M, Bunting MJ, et al. (2021) Linking modern pollen accumulation rates to biomass: Quantitative vegetation reconstruction in the western Klamath Mountains. The Holocene. https://doi.org/10.1177/0959683620988038.

Kroeber T (1959) The Inland Whale. University of California Press.

McCune B, Grace JB, Urban DL (2002) Analysis of ecological communities (Vol. 28). Gleneden Beach, OR: MjM software design.

Niinemets Ü, Valladares F (2006) Tolerance to shade, drought, and waterlogging of temperate Northern Hemisphere trees and shrubs. Ecological Monographs 76:521–547.

Pearsall CE, Pearsall HM, Neall HL (1928) History and genealogy of the Pearsall family in England and America. San Francisco: Printed by H.S. Crocker Co.

Polanco-Martinez JM (2020) RolWinMulCor: An R package for estimating rolling window multiple correlation in ecological time series. Ecological Informatics 60. https://doi.org/10.1016/j.ecoinf.2020.101163

Riley-Thron KL (2001) Forest, Fire, Home: Experiences with wildfire in the Klamath Mountains of Northern California. Thesis, Humboldt State University.
Schenk SM, Gifford EW (1952) Karok ethnobotany. *Anthropological Record* 13:377–92.
Stewart WC, Sharma B (2015) Carbon calculator tracks the climate benefits of managed private forests. California Agriculture. January-March: 21-26.
Telford R (2013) Running correlations - running into problems(2013). URL https://quantpalaeo.wordpress.com/2013/01/04/running-correlations-running-into-problems/.
Warburton AD, Endert JF (1966) Indian Lore of the North California Coast. Pacific Pueblo Press, Santa Clara, CA.
Waterman TT (1920) Yurok Geography. Publication in American Archaeology and Ethnology, Vol. 16, No. 5, pages: 177-315. University of California Press, Berkeley, Ca. Reprint 1993. Trinidad Museum Society.
